# Understanding Australian adults’ preferences for setting goals to reduce unhealthy food and beverage intake: a cross-sectional study

**DOI:** 10.1017/jns.2025.10022

**Published:** 2025-07-16

**Authors:** Chelsea E. Mauch, Ashlee Fuchs, Caitlin A. Howlett, Gilly A. Hendrie

**Affiliations:** 1 Human Health, Health and Biosecurity, Commonwealth Scientific and Industrial Research Organisation (CSIRO), SAHMRI, Adelaide 5000, SA, Australia; 2 Nutrition and Dietetics, College of Nursing and Health Sciences, Flinders University, Bedford Park 5042, SA, Australia

**Keywords:** Adult, Behaviour change, Discretionary food, Goal setting, Nutrition, ADG, Australian Dietary Guidelines, ADHE, Australian Guide to Healthy Eating, BCT, Behaviour Change Technique, BFI, Big Five Inventory, BMI, Body Mass Index, CSIRO, Commonwealth Scientific Industrial Research Organisation, WHO, World Health Organisation

## Abstract

Overconsumption of unhealthy, discretionary, foods and beverages are associated with an increased risk of weight gain and non-communicable diseases, including diabetes, heart disease, and cancer. This cross-sectional study explored preferences for setting goals to reduce discretionary food and beverage consumption. The online survey included items about discretionary food and beverage intake, goal setting preferences to reduce intake, habit strength, personality traits, and demographic characteristics. A total of 2664 Australian adults completed the survey. The sample was mostly female (65.9%), half (52.8%) were aged between 30–49 years, and the median intake of discretionary food and beverages was 4.9 (IQR: 3.6 to 7.2) serves per day. Multinomial logistic regression and ordinal logistic regression models were used to explore demographic and psychological predictors of the helpfulness of long-term and short-term goals, elimination and gradual goals, specific food goals, specific eating occasion and food goals, self-set goals, collaboratively set goals, and assigned goals. The results showed participants with higher habit strength had greater odds of finding short-term (OR 1.40, 95% CI 1.06–1.86), gradual (OR 1.14, 95% CI 1.01–1.29), specific (OR 1.35, 95% CI 0.84–1.76), assigned (OR 1.38, 95% CI 1.14–1.66) and collaborative goals (OR 1.24, 95% CI 1.01–1.53) helpful. The results also indicated that age and gender were important predictors of goal setting preferences, particularly for long-term goals, elimination goals, broad goals, and collaborative goals. Interventions to reduce discretionary food and beverage intake are needed and consideration of goal setting preferences could be a novel way to developing more tailored and effective dietary interventions.

## Background

Obesity is a significant, but largely preventable, global public health challenge.^(1)^ The worldwide prevalence of obesity has almost tripled since 1975,^(1)^ with prevalence rates expected to escalate to 1 billion adults by 2025 if increasing trends cannot be curbed.^(2)^ According to data from the Australian Bureau of Statistics, overweight and obesity affects 12.5 million Australian adults (or 67% of the adult population),^(3)^ making obesity prevention and treatment a priority.

Poor diet quality is a leading risk factor for premature mortality^(4)^ and remains a key modifiable risk factor for the development of obesity.^(5)^ Findings from the Commonwealth Scientific Industrial Research Organisation (CSIRO) Healthy Diet Score indicate that Australians dietary patterns do not comply with the Australian Guide to Healthy Eating (AGHE), with discretionary foods and beverages being the worst performing area of diets for Australian adults.^(6)^ Discretionary foods and beverages are those that provide little nutritional value, and are typically high in saturated fat, added sugars, added salt and/or alcohol.^(7)^ Examples include cakes and biscuits, sugar-sweetened beverages, processed meats, confectionary and alcoholic beverages.^(7)^ The AGHE states that discretionary foods and beverages are not an essential component of a healthy diet and recommends limiting the consumption of these foods to ‘sometimes and in small amounts’.^(8)^

In 2011–12, discretionary foods and beverages contributed to approximately one third of total daily energy intake in Australian adults.^(7)^ Similarly, in the United States, 80% of people aged 71 years and over, and 90% of all other age groups are exceeding dietary guidelines for both saturated fats and sugar – nutrients that are found in large quantities in many discretionary foods and beverages.^(9)^ Discretionary foods and beverages can displace healthy foods from the five core food groups, such as wholegrains, fruits and vegetables, and due to their energy density may increase the risk of weight gain.^(10)^ The intake of discretionary foods and beverages is associated with an increased risk of non-communicable diseases such as diabetes, heart disease and cancer.^(11)^ Previous public health nutrition interventions targeting obesity and chronic disease prevention have resulted in small-to-moderate improvements in health behaviours.^(12)^ Therefore, opportunities remain to develop more effective interventions to improve population dietary intake through a reduction in discretionary foods and beverages.

Goal setting has been shown to be an effective behaviour change technique (BCT) for improving energy balance behaviours such as dietary intake and physical activity.^(13–15)^ A goal can be described as something an individual is consciously trying to achieve, with a future end-state in mind.^(16)^ Goal setting has been widely researched in psychology, with goal setting theory recommending that goals be both specific and challenging.^(17)^ Goal setting also appears to be more effective when paired with feedback regarding the outcomes of the behaviour.^(17)^

While goal setting has been shown to be effective for changing dietary behaviours such as increasing fruit and vegetable intake, currently there is limited literature exploring the effectiveness of goal setting in relation to reducing discretionary food and beverage consumption.^(14)^ Goals targeting an increase in a positive behaviour, for example, ‘eating one extra serve of fruit per day’, are referred to as approach goals,^(18)^ while goals targeting a decrease in a negative behaviour, for example, ‘avoid sweet treats’, are referred to as avoidance goals.^(18)^ Approach goals tend to be well structured, measurable and have a clear end point, whereas avoidance goals can lack direction, focus on avoiding undesirable behaviours, and progress toward these goals can be difficult to monitor.^(18,19)^ However, avoidance goals that are more specific, for example, ‘eating one less serve of chocolate per day’ have been shown to be more effective than broad avoidance goals.^(18)^ Therefore, a greater understanding of how to set and use goals with an ‘eat less’ motive is needed.

The effectiveness of goal setting as a BCT is dependent on multiple factors, including the type of goal, target behaviour and individual characteristics.^(20)^ Research in psychology has identified demographics and personality traits as key contributors to attitudes towards behaviour change, which is further outlined and supported by the Theory of Planned Behaviour.^(20)^ Additionally, evidence highlights that consumption of discretionary food and beverages differs by demographic characteristics such as age, gender, and education level.^(21,22)^ There is also evidence to suggest that personality traits and habits are associated with dietary intake.^(23,24)^ Understanding whether personality traits, habit strength, and demographic characteristics are associated with variations in goal setting preferences may allow for more tailored and effective goal setting strategies to be developed for public health nutrition interventions.

This exploratory study aimed to assess the goal setting preferences of Australian adults in relation to reducing discretionary food and beverage intake and to determine if preferences differed between subgroups of the population, including by demographic characteristics, habit strength and personality traits. The application of the findings of this study will be used to inform interventions to reduce discretionary food intake, therefore the analysis was limited to those with discretionary food intakes that were higher than the recommended intakes.

## Methods

### Study design and recruitment

This cross-sectional study explored preferences for various goal setting parameters related to reducing discretionary food and beverage intake. A survey was administered through Alchemer, an online survey platform, between October and December 2021. Participants were recruited via an email invitation to a database of individuals who had participated in CSIRO health and nutrition research and who had consented to being contacted about future research, as well as through Facebook advertisements. Upon completion of the survey, participants were invited to enter a draw to win one of seven $50 grocery vouchers. The study was approved by CSIRO’s Human Research Ethics Committee (approval no. 2021_097_LR) and Flinders University Human Research Ethics Committee (approval no. 4908).

### Participants

Participants were eligible if they were currently living in Australia and aged 18 years or older. Those with extreme values for weight (less than 13kg or greater than 250kg), height (less than 1 metre or greater than 3 metres), and Body Mass Index (BMI) (less than 13kg/m^2^ or greater than 97kg/m^2^) were excluded. Participants classified as underweight (BMI less than 18.5kg/m^2^) were also excluded from the main analyses as they generally would not be advised to reduce their dietary intake.

### Survey design

The online survey included questions relating to discretionary food and beverage intake, goal setting, habit strength, personality traits, and demographic characteristics. A copy of the survey tools/questionnaire is available in the Supplementary Material (see Supplemental File 1).

### Dietary intake

Items from the CSIRO Healthy Diet Score survey were used to estimate discretionary food and beverage intake. Details of the development and validation of the CSIRO Healthy Diet Score survey has been described elsewhere.^(6,25)^ Intake was reported as a frequency (daily, weekly, monthly) and standard portions across 11 categories of discretionary food and beverages, including alcohol and sugar sweetened beverages, chocolate/confectionary, cakes/biscuits, processed meat products, savoury snacks, muesli and snack bars, ice cream, fried potato products and savoury pies and pastries. Responses were used to calculate serves per day. Adjustment factors were applied to account for self-reporting bias, which is usually in the direction of under-reporting for discretionary foods and beverages.^(26)^ Adjustment factors have been previously developed and are based on a ratio (usual portion size estimated from national data to standard serving size from Australian Dietary Guidelines). When applied to self-reported data, adjustment factors improve estimates of food group intake.^(26)^ Individuals’ estimated daily intake was compared to the age and sex specific recommendations in the Australian Dietary Guidelines (ADGs).^(6,27)^

### Goal setting

In this study, goal setting was focused on reducing discretionary food intake. A series of questions assessed frequency of attempts to reduce discretionary food and beverage intake (‘I haven’t ever tried’, ‘1–2 times’, ‘3–4 times’, ‘5–6 times’, ‘7 or more times’), perceived level of success (‘not very successful at all’, ‘not successful’, ‘somewhat successful’, ‘successful’ or ‘very successful’) and use of goal setting to do so (‘never’, ‘occasionally’, ‘sometimes’, ‘often’, or ‘always’).

To assess preferences for various parameters around goal setting to reduce discretionary food and beverage intake, participants were asked a series of questions and responded using a 5-point Likert scale with the following response options: ‘not at all helpful’, ‘slightly helpful’, ‘somewhat helpful’, ‘very helpful’, or ‘extremely helpful’. The key goal setting parameters were guided by the literature, and included goal proximity (short-term and long-term goals), specificity (broad all food goals, specific food-type goals and specific eating occasion goals), difficulty (elimination goals and gradual reduction goals) and level of support (self-set goals, collaboratively set goals, and assigned goals).^(13,28)^ Finally, the open-ended question, ‘Regardless if you have set a goal in the past or not, describe what a goal for eating less discretionary food might look like for you?’ was included to explore participants ability to set ‘eat less’ goals and identify aspects of goal setting that may require further consideration.

### Habit strength

The 12-item Self-Report Habit Index was used to assess habit strength relating to discretionary food and beverage intake (e.g., ‘Eating discretionary food and beverages is something I do frequently’).^(29)^ The questionnaire asks participants to indicate their level of agreement on a five-point Likert scale from 1 (strongly disagree) to 5 (strongly agree), with higher scores reflecting stronger habit strength.

### Personality traits

The Big Five taxonomy, which consists of personality traits, including agreeableness, neuroticism, openness to experience, conscientiousness and extraversion, is the most influential model for explaining how individuals differ on dimensions of personality.^(30,31)^ The Big Five Inventory (BFI)^(32)^ is a validated self-reported questionnaire that is used to assess the aforementioned traits. The BFI-10 is a brief 10-item version of the BFI and has been validated for instances where time is limited, or where administering the BFI-44 would be impractical or burdensome.^(32)^ Items are assessed using a Likert scale ranging from ‘strongly disagree’ to ‘strongly agree’, with higher scores indicating a greater expression of a personality trait.

### Demographics

Demographic items included age (measured in five-year age categories), gender (male, female, non-binary, gender not listed), country of birth (Australia or other), level of education (less than high school, finished high school, certificate level, diploma level, bachelor’s degree, post graduate degree) and self-reported height (metres) and weight (kilograms).

### Data preparation

Survey responses from Alchemer (https://www.alchemer.com/) were imported directly into IBM SPSS Statistics 27 for cleaning and analysis. Serves of each discretionary food and beverage category consumed was determined from portion and frequency questions, and intake of each category summed to estimate a total daily discretionary intake (in serves). Extreme outliers were defined as participants consuming at least 2.75 times their basal metabolic rate^(33)^ in energy from discretionary foods and beverages, assuming a physical activity level of 1.55.^(34)^ These extreme reporters were excluded from analysis. Also, given the focus on goal setting to reduce discretionary food intake, the analysis was limited to those with intakes that were higher than the recommendations in the Australian Dietary Guidelines.

The mean of the 12 items of the Self-Report Habit Index was calculated. Five of the items on the BFI-10 were reverse scored before summing with the corresponding personality trait item to produce a score out of ten for each trait, with a higher score reflecting a greater expression of that trait. Gender categories ‘non-binary’ and ‘gender not listed’ were excluded in goal setting regression models due to small sample sizes, which violated statistical assumptions. Height (metres) and weight (kilograms) were used to calculate BMI (kg/m^2^); the World Health Organisation (WHO) cut-offs were used for classification of weight status. Age was collapsed into four broader age categories: 18–29 years, 30–49 years, 50–69 years and ≥70 years to more closely resemble age categories of the ADGs.^(27)^ Education data was collapsed into a dichotomous variable (‘University educated’ and ‘Not university educated’) due to the small sample sizes of some categories.

### Statistical analysis

Descriptive statistics (i.e., frequency and percent, and median and interquartile range (IQR) were calculated to describe participant characteristics, goal setting preferences related to eating less discretionary food and total daily discretionary intake (serves/day). Cumulative odds ordinal logistic regressions were used to explore the helpfulness of long-term, gradual, broad, and self-set goals to reduce discretionary food intake. The assumption of proportional odds, measured using the test of parallel lines, was violated for the remaining goal setting parameters under exploration. For those goal setting parameters, stepwise multinomial logistic regressions were conducted. For ease of interpretation of results, the five-point helpfulness scale of goal setting parameters to reduce discretionary food intake was collapsed into three categories (*not at all helpful*, *slightly or somewhat helpful* and *very or extremely helpful*) for the multinomial logistic regressions. The dependent variable for each regression model was a different goal setting parameter, for example, how helpful are short-term goals to reduce discretionary food intake. Independent variables included gender (reference category: *male*), age group (reference category: *70+ years*), highest level of education (reference category: *no university*), BMI (kg/m^2^), total discretionary intake (serves/day), habit strength (mean score out of 5), neuroticism score, openness score, conscientiousness score, agreeableness score and extraversion score (score out of 10 for each personality item). Ordinal regression parameter estimates were converted into odds ratios and 95% confidence intervals. Results were considered statistically significant if *p* <0.05.

The results of the multinomial regression analyses were similar for *not at all helpful* vs *very or extremely helpful*, and *not at all helpful* vs *slightly or somewhat helpful*. Therefore, for simplicity, we have focused the results on the two extremes of *not at all helpful* vs *very or extremely helpful*. The results for *not at all helpful* vs *slightly or somewhat helpful* are available in the Supplementary Material (Supplemental File 2).

## Results

### Participant characteristics

A total of 2664 participants completed the online survey (Table 1). Almost half of the sample was aged between 30 and 49 years (48.4%), and 4.1% was aged 70 years or older. Most participants were female (70.1%), born in Australia (83.6%), university educated (68.5%), and classified as individuals living with overweight or obesity (62.6%). The median reported discretionary food and beverage intake was 3.74 (IQR: 2.17–5.97) serves per day. Of the total sample, 68.9% exceeded the age and sex specific ADGs recommendation for discretionary food and beverage intake and were also classified as healthy weight or above. Their median intake of discretionary foods and beverages was 4.9 (IQR: 3.6–7.2) serves per day. Demographic characteristics of those exceeding the ADGs recommendations were similar to the total sample, with the exception of a slightly greater proportion of males (32.8% versus 28.7%), people aged between 30 and 49 years (52.8% versus 48.4%), and people with obesity (34.2% versus 29.8%). The subsample of ‘over-consumers’ of discretionary foods was the focus of the analysis.

**Table 1.** Characteristics of full sample, and sample exceeding Australian Dietary Guidelines for discretionary food and beverage intake

Demographic variable	Total (n=2664)		Sample exceeding Australian Dietary Guidelines (n=1835^a^)	
	n	%	n	%
Total	2664	100.0	1835	100.0
Gender				
Male	765	28.7	602	32.8
Female	1867	70.1	1210	65.9
Non-binary	23	0.9	18	1.0
Gender not listed	9	0.3	5	0.3
Age group				
18–29 years	411	15.4	301	16.4
30–49 years	1289	48.4	969	52.8
50–69 years	856	32.1	514	28.0
70 + years	108	4.1	51	2.8
Highest level of education				
High school, Trade, TAFE or diploma	838	31.5	621	33.9
University	1826	68.5	1214	66.2
Country of birth				
Australia	2227	83.6	1552	84.6
Overseas	437	16.4	283	15.4
Weight status				
Underweight	51	1.9	NA	NA
Healthy weight	946	35.5	589	32.1
Overweight	873	32.8	618	33.7
Obesity	794	29.8	628	34.2
	Median	IQR	Median	IQR
Discretionary intake (adjusted serves/day)	3.74	2.17–5.97	4.90	3.60–7.16
Habit strength (max 5)^b^	3.17	2.58–3.75	3.42	2.92–3.83
Extraversion (max 10)^b^	6.00	4.00–6.00	6.00	4.00–7.00
Agreeableness (max 10)^b^	7.00	6.00–8.00	7.00	6.00–8.00
Conscientiousness (max 10)^b^	7.00	6.00–8.00	7.00	6.00–8.00
Neuroticism (max 10)^b^	6.00	5.00–8.00	6.00	5.00–8.00
Openness (max 10)^b^	7.00	6.00–8.00	7.00	6.00–8.00

Abbreviations. n, sample size; TAFE, Technical and Further Education; NA, not available; IQR, interquartile range;

a Excludes underweight participants;

b Higher scores indicate stronger habit strength or more of the personality trait.

### Goal setting helpfulness

One in three participants reported that short-term goals were *very or extremely helpful* and 35.9% reported that gradual goals were *very or extremely helpful*. Seven percent of participants reported that short-term goals were *not at all helpful* compared with 13% for long-term goals. Almost half of respondents considered elimination goals to be *not at all helpful* (49.9%) (Table 2). There was a preference for more specific goals, such as those targeting specific types of food (44.6% *very or extremely helpful*) or eating occasions (38.2% *very or extremely helpful*), over more general goals targeting all types of discretionary foods and beverages (22.5% *very or extremely helpful*). Participants also reported that collaborative and self-set goals were more helpful than assigned goals, with 26% reporting that assigned goals were *not at all helpful*.

**Table 2.** Goal setting helpfulness for measures of proximity, difficulty, specificity, and level of support amongst the sample exceeding dietary guidelines for discretionary food and beverage intake (n=1835)

Goal setting parameter	Not at all helpful	Slightly helpful	Somewhat helpful	Very helpful	Extremely helpful
	n (%)				
Proximity					
Short-term goals	128 (7.0)	374 (20.4)	707 (38.5)	514 (28.0)	112 (6.1)
Long-term goals	240 (13.1)	510 (27.8)	672 (36.6)	329 (17.9)	84 (4.6)
Difficulty					
Gradual goals	218 (11.9)	393 (21.4)	566 (30.8)	526 (28.7)	132 (7.2)
Elimination goals	916 (49.9)	331 (18.0)	280 (15.3)	201 (11.0)	107 (5.8)
Specificity					
Food type goals	135 (7.4)	298 (16.2)	583 (31.8)	643 (35.0)	176 (9.6)
All food goals	267 (14.6)	509 (27.7)	646 (35.2)	308 (16.8)	105 (5.7)
Eating occasion goals	184 (10.0)	337 (18.4)	613 (33.4)	576 (31.4)	125 (6.8)
Level of support					
Assigned goals	478 (26.0)	392 (21.4)	441 (24.0)	368 (20.1)	156 (8.5)
Collaborative goals	85 (4.6)	370 (20.2)	682 (37.2)	539 (29.4)	159 (8.7)
Self-set goals	279 (15.2)	322 (17.5)	494 (26.9)	510 (27.8)	230 (12.5)

Abbreviations. n, sample size.

### Predictors of helpfulness of goal setting parameters

Multinomial and ordinal regression analyses were used to explore predictors of helpfulness of the various goal setting parameters. All regression models showed good model fit. The focus of the discussion of the comparison was on *not at all helpful* vs *very or extremely helpful*. Full models are provided in the Supplementary Material (Supplementary File 3).

#### Proximity of goals

Males and younger adults were most likely to find long-term goals helpful. The odds of males finding long-term goals helpful was 1.41 (95% CI 1.17–1.70) times higher than that of females, and the odds of 30–49 and 50–69-year-olds finding long-term goals helpful was 1.81 (95% CI 1.08–3.05) and 2.34 (95% CI 1.38–3.96) times higher than older adults aged ≥70 years. Compared to having a university degree, those without a degree had higher odds of finding short-term goals *very of extremely helpful* (OR 1.92, 95% CI 1.23–2.97). Participants with higher habit strength had higher odds of finding short-term goals *very or extremely helpful* than *not at all helpful* (OR 1.40, 95% CI 1.06–1.86), and had lower odds of finding long-term goals helpful (OR 0.84, 95% CI 0.74–0.95, see Table 3). Participants that scored higher on extraversion (OR 0.80, 95% CI 0.69–0.93) and openness (OR 0.73, 95% CI 0.63–0.85) have lower odds of finding short-term goals *very or extremely helpful*.

**Table 3.** Logistic regression investigating predictors of short-term and long-term goal helpfulness

	Short-term goals^a^ (Not at all vs. very or extremely helpful)				Long-term goals^b^			
Variables	OR	95% CI			OR	95% CI		
		Lower	Upper	p-value		Lower	Upper	p-value
Intercept				.044				
Male (ref: female)	0.71	0.47	1.09	.119	1.41	1.17	1.70	.000***
Age 18–29 (ref:70+)	2.06	0.61	6.91	.245	1.62	0.93	2.81	.088
Age 30–49 (ref:70+)	2.43	0.78	7.63	.127	1.81	1.08	3.05	.025*
Age 50–69 (ref:70+)	1.83	0.59	5.75	.298	2.34	1.38	3.96	.002**
No uni (ref: Uni)	1.92	1.23	2.97	.004**	1.18	0.98	1.41	.080
BMI	0.98	0.95	1.00	.074	0.99	0.98	1.01	.289
Discretionary intake	0.97	0.92	1.01	.130	0.98	0.96	1.01	.140
Habit strength	1.40	1.06	1.86	.018*	0.84	0.74	0.95	.005**
Extraversion	0.80	0.69	0.93	.003**	0.95	0.89	1.02	.150
Agreeableness	1.18	1.04	1.34	.013*	1.05	1.00	1.12	.072
Conscientiousness	1.07	0.94	1.23	.288	1.02	0.96	1.08	.575
Neuroticism	0.94	0.85	1.05	.291	0.94	0.90	0.99	.012*
Openness	0.73	0.63	0.85	.000***	0.96	0.90	1.02	.157

Abbreviations. B, beta; SE, standard error; OR, odds ratio; CI, confidence interval; ref, reference; BMI, body mass index;

a Multinomial logistic regression;

b Ordinal logistic regression;

* p<0.05; **p<0.01; ***p<0.001.

#### Difficulty of goals

Males had greater odds of finding elimination goals *very or extremely helpful* (OR 2.26, 95% CI 1.70–3.02) than females (Table 4). The odds of those without a university degree finding elimination goals *very or extremely helpful* relative to *not at all* was 1.47 times higher than those with a university degree (OR 1.47, 95% CI 1.11–1.94) and had greater odds of finding gradual goals helpful (OR 1.30, 95% CI 1.09–1.56). Higher habit strength, conscientiousness, and neuroticism were also associated with greater odds of finding gradual goals helpful (OR 1.14, 95% CI 1.01–1.29; OR 1.07, 95% CI 1.01–1.14; and OR 1.06, 95% CI 1.02–1.11, respectively).

**Table 4.** Logistic regression investigating predictors of elimination and gradual goal helpfulness

	Elimination goals^a^ (Not at all vs. very or extremely helpful)				Gradual goals^b^			
Variables	OR	95% CI			OR	95% CI		
		Lower	Upper	p-value		Lower	Upper	p-value
Intercept				.046				
Male (ref: female)	2.26	1.70	3.02	.000***	1.00	0.83	1.20	.997
Age 18–29 (ref:70+)	0.96	0.37	2.50	.940	1.72	0.99	2.96	.053
Age 30–49 (ref:70+)	1.34	0.55	3.28	.526	1.16	0.69	1.93	.583
Age 50–69 (ref:70+)	1.78	0.72	4.39	.209	1.20	0.71	2.02	.493
No uni (ref: Uni)	1.47	1.11	1.94	.007**	1.30	1.09	1.56	.004**
BMI	1.00	0.98	1.02	.872	1.00	0.99	1.02	.769
Discretionary intake	1.02	0.99	1.05	.269	1.00	0.98	1.02	.966
Habit strength	0.94	0.77	1.14	.519	1.14	1.01	1.29	.033*
Extraversion	1.02	0.92	1.13	.660	1.00	0.94	1.07	.904
Agreeableness	1.02	0.93	1.11	.736	1.05	0.99	1.11	.131
Conscientiousness	1.02	0.93	1.12	.720	1.07	1.01	1.14	.014*
Neuroticism	0.96	0.89	1.04	.303	1.06	1.02	1.11	.010*
Openness	1.03	0.94	1.13	.552	0.98	0.92	1.04	.518

Abbreviations. B, beta; SE, standard error; OR, odds ratio; CI, confidence interval; ref, reference; BMI, body mass index;

a Multinomial logistic regression;

b Ordinal logistic regression;

* p<0.05; **p<0.01; ***p<0.001.

#### Specificity of goals

Males were more likely to find food goals and broad goals more helpful than females. The odds of males finding specific food related type goals *very or extremely helpful* was 1.66 times higher (OR 1.66, 95% CI 1.07–2.58) than that of females, whereas the odds of finding broad goals helpful was 1.47 times higher for males (OR 1.47, 95% CI 1.22–1.77) than for females (see Table 5). The odds of those without a university degree finding broad goals helpful was 1.31 times higher than those with a university degree (95% CI 1.09–1.57) and the odds of 30–49 and 50–69-year-olds finding broad goals helpful was 1.85 (95% CI 1.10–3.12) and 2.22 times higher (95% CI 1.31–3.75) than those aged ≥70 years. Younger adults (18–29 and 30–49-year-olds) had higher odds of finding eating occasion goals *very or extremely helpful* (OR 3.79, 95% CI 1.49–9.60; and OR 2.48, 95% CI 1.08–5.69, respectively) than older adults aged ≥70 years. In addition, those who scored higher on openness had lower odds of finding eating occasion goals *very or extremely helpful* (OR 0.80, 95% CI 0.71–0.91), whereas those who scored higher on habit strength had greater odds of finding specific goals *very or extremely helpful* (OR 1.35, 95% CI 1.04–1.76).

**Table 5.** Logistic regression investigating predictors of food type goal (i.e., specific goal), all food goals (i.e. broad goals), and eating occasion goal helpfulness

	Food type goals i.e., specific goals (Not at all vs. very or extremely helpful)^a^				All food goals i.e., broad goals^b^				Eating occasion goals (Not at all vs. very or extremely helpful)^a^			
Variables	OR	95% CI		p-value	OR	95% CI		p-value	OR	95% CI		p-value
		Lower	Upper			Lower	Upper			Lower	Upper	
Intercept				.488								.062
Male (ref: female)	1.66	1.07	2.58	.024*	1.47	1.22	1.77	.000***	0.70	0.49	1.01	.057
Age 18–29 (ref:70+)	4.04	1.39	11.73	.010*	1.60	0.93	2.78	.092	3.79	1.49	9.60	.005**
Age 30–49 (ref:70+)	2.52	1.00	6.35	.050	1.85	1.10	3.12	.020*	2.48	1.08	5.69	.032*
Age 50–69 (ref:70+)	1.43	0.57	3.59	.444	2.22	1.31	3.75	.003**	1.94	0.85	4.45	.118
No uni (ref: Uni)	0.72	0.48	1.06	.097	1.31	1.09	1.57	.004**	0.88	0.62	1.25	.481
BMI	1.00	0.97	1.03	.990	1.00	0.99	1.02	.455	1.00	0.98	1.03	.929
Discretionary intake	0.96	0.92	1.00	.065	0.98	0.96	1.00	.117	0.99	0.95	1.03	.677
Habit strength	1.35	1.04	1.76	.023*	0.89	0.79	1.01	.063	1.04	0.82	1.32	.761
Extraversion	0.97	0.84	1.11	.629	0.97	0.91	1.03	.296	0.88	0.78	1.00	.056
Agreeableness	1.00	0.88	1.14	.987	1.00	0.95	1.06	.975	1.10	0.98	1.23	.114
Conscientiousness	0.99	0.87	1.13	.887	0.99	0.93	1.04	.601	1.02	0.91	1.14	.790
Neuroticism	1.08	0.97	1.19	.168	1.00	0.96	1.05	.869	0.98	0.89	1.07	.599
Openness	0.89	0.77	1.01	.077	0.99	0.93	1.05	.684	0.80	0.71	0.91	.001**

Abbreviations. B, beta; SE, standard error; OR, odds ratio; CI, confidence interval; ref, reference; BMI, body mass index;

a Multinomial logistic regression;

b Ordinal logistic regression;

* p<0.05; **p<0.01; ***p<0.001.

#### Level of support

Younger adults had greater odds of finding all three methods of goal setting (assigned, self-set, and collaborative) more helpful than adults aged ≥70 years, but the most marked difference was for collaborative goal setting. The odds of younger adults finding collaborative goals helpful was 15.25 times larger than adults aged ≥70 years finding these types of goals helpful (OR 15.25, 95% CI 5.66–41.08 in 18–29-year-olds), and the odds were also higher for 30–49-year-olds (OR 7.48, 95% CI 3.03–18.46) and 50–59-year-olds (OR 4.00, 95% CI 1.62–9.88). Those with higher BMI and habit strength had lower odds of finding self-set goals helpful (OR 0.98, 95% CI 0.97–0.99 and OR 0.73, 95% CI 0.64–0.82, respectively). Participants with higher habit strength had greater odds of considering assigned goals (OR 1.38, 95% CI 1.14–1.66) and collaborative goals (OR 1.24, 95% CI 1.01–1.53) *very or extremely helpful* (Table 6).

**Table 6.** Logistic regression investigating predictors of assigned goal, self-set goal, and collaborative goal helpfulness

	Assigned goals (Not at all vs. very or extremely helpful)^a^				Self-set goals^b^				Collaborative goals^a^			
Variables	OR	95% CI		p-value	OR	95% CI		p-value	OR	95% CI		p-value
		Lower	Upper			Lower	Upper			Lower	Upper	
Intercept				.003								.020
Male (ref: female)	1.22	0.92	1.63	.170	1.23	1.02	1.49	.031*	0.98	0.71	1.35	.894
Age 18–29 (ref:70+)	5.41	2.01	14.52	.001**	1.89	1.08	3.29	.025*	15.25	5.66	41.08	.000***
Age 30–49 (ref:70+)	3.51	1.37	8.96	.009**	1.83	1.09	3.10	.023*	7.48	3.03	18.46	.000***
Age 50–69 (ref:70+)	2.66	1.03	6.83	.042*	2.04	1.20	3.47	.009**	4.00	1.62	9.88	.003**
No uni (ref: Uni)	1.20	0.91	1.57	.197	0.90	0.75	1.09	.282	1.09	0.80	1.48	.572
BMI	0.99	0.97	1.01	.481	0.98	0.97	0.99	.001**	1.02	1.00	1.04	.091
Discretionary intake	1.01	0.98	1.05	.409	0.99	0.97	1.01	.396	0.98	0.95	1.02	.392
Habit strength	1.38	1.14	1.66	.001**	0.73	0.64	0.82	.000***	1.24	1.01	1.53	.037*
Extraversion	1.01	0.91	1.11	.916	0.98	0.92	1.05	.584	0.97	0.87	1.08	.570
Agreeableness	1.13	1.04	1.23	.006**	1.06	1.00	1.12	.070	1.09	0.99	1.20	.074
Conscientiousness	0.92	0.85	1.01	.072	1.08	1.02	1.14	.013*	0.96	0.87	1.06	.423
Neuroticism	1.05	0.98	1.13	.149	0.94	0.89	0.98	.006**	1.06	0.98	1.15	.145
Openness	1.01	0.92	1.11	.825	1.05	0.99	1.12	.094	0.98	0.88	1.08	.658

Abbreviations. B, beta; SE, standard error; OR, odds ratio; CI, confidence interval; ref, reference; BMI, body mass index;

a Multinomial logistic regression;

b Ordinal logistic regression;

* p<0.05; **p<0.01; ***p<0.001.

## Discussion

The aim of this exploratory, cross-sectional study was to explore the goal setting preferences of Australian adults to support a reduction in discretionary food and beverage consumption and determine whether goal setting preferences differed by demographic and personality characteristics. This study was the first of its kind to examine individual preferences for goal setting in relation to reducing discretionary food and beverage intake. Moreover, few studies have explored preferences for goal setting parameters in relation to demographic characteristics. Overall, our findings suggest that individuals’ strength of habits as well as certain demographic characteristics, such as age and gender, play an important role in shaping their goal setting preferences. Age and gender were the most consistent predictors of goal setting preference, particularly for long-term goals, elimination goals, broad goals, and collaborative goals. A deeper understanding of what types of goals subgroups of the population prefer in relation to the structure of dietary behaviour change goals will allow for greater tailoring in the design of new interventions.

Habits are behaviours performed with a high degree of frequency and automaticity and are usually triggered by cues within a stable context.^(35)^ In this large sample of Australian adults, we found that stronger habit strength was associated with preferences for setting short-term, more gradual, specific, and supported (i.e., either assigned or collaborative) goals for reducing discretionary food intake. This suggests that those who have stronger habits may require ongoing support and do better with short-term goals that are achieved more gradually and targeted at specific foods types to overcome habitual eating behaviours. Although goal setting theory emphasises that goals should be difficult in nature, it is equally important that goals are achievable to facilitate commitment and promote changes in behaviour.^(16)^ Preferences for goal difficulty is influenced by personal factors such as self-efficacy.^(36)^ Therefore, when setting dietary goals within interventions, it is important to consider an appropriate level of goal difficulty because setting goals that are too difficult may weaken perceived self-efficacy and lead to eventual goal failure. On the other hand, goals that are too easy may lead to boredom and/or result in smaller dietary change.^(36)^ This indicates that tailoring goal difficulty could be an important part of intervention success and that during the initial stages of goal setting, individuals should be supported and guided to reflect on their own abilities and skills to develop goals with an appropriate degree of difficulty. While goal setting is important for behaviour change generally,^(37)^ the type of goals that individuals prefer or that are effective may differ for different health behaviours. For example, research investigating smoking cessation found smokers who set specific, long-term and ambitious goals such as ‘never smoke again’ were more likely to succeed in quitting than those who set short-term, non-specific and less ambitious goals such as ‘see how I go’.^(38)^ This suggest that our results around ‘eat less’ dietary goals may not be generalisable to other health-related behaviours. Given that discretionary foods are the poorest performing area of diet quality, with Australian adults eating twice as much as is recommended^(39)^ and that eating behaviours are largely habitual,^(40)^ it seems imperative to tailor goals within individual level interventions to align with individual goal setting preferences, particularly for those who have stronger habitual eating behaviours.

The findings also revealed that age and gender were the most consistent predictors of goal setting preference, particularly for long-term goals, elimination goals, broad goals, and collaborative goals. We found that people aged between 30 and 69 preferred broad and long-term goals more than people aged over 70 years. There is limited literature that has explored goal setting preferences in relation to age. However, previous research has found that diet quality increases with increasing age, which may reflect increased health consciousness.^(41)^ Our findings are also consistent with a qualitative study that explored goal setting and lifestyle behaviours for people aged over 50 years and found that some people became more motivated to age healthily, suggesting that, to some degree, longer-term goals associated with healthy aging may resonate with adults over 50 years.^(42)^ People in this age group also reported a preference for self-set and collaborative goals, which may reflect higher self-efficacy and a desire to work together, compared to older adults who may start to lose confidence as they begin to face cognitive decline.^(43)^ Younger adults (18–29 years) also found collaborative goals helpful, and a preference towards structured goal setting – that is, goals that were assigned and set around specific eating occasions goals. These preferences may link to a desire to have external accountability or stronger guidance to help in resisting temptations for discretionary foods when socialising with others.^(44)^ There were also some differences in goal preferences by gender and education level. Compared to females, men appeared to find broad, long-term and elimination goals helpful. Given men tend to have poorer diet quality and consume more discretionary foods,^(6)^ it is important that we understand the kind of goals that would be appealing and effective for men to change their dietary habits. Our findings also revealed that education level can influence the type of goals people prefer to set. For instance, those with lower education level preferred short term and elimination goals. Low education attainment is associated with lower food literacy, nutrition knowledge and less healthy eating habits.^(45)^ As a result, our findings suggest that clear and actionable behaviour change messages might be more effective in this group. Tailoring goal-based interventions to segments of the population might help to provide messages and strategies that are more aligned with individuals based on their known preferences for goal settings. This is worthwhile piloting in future intervention research.

Despite public health efforts to improve diet quality, discretionary food intake remains persistently high. There is growing evidence that overconsumption of highly processed, discretionary foods is positively associated with increased risk of weight gain and obesity and poorer health outcomes.^(1)^ Population dietary guidelines provide advice to improve eating habits but take a ‘one-size-fits-all’ approach. Further, mass media lifestyle changes such as ‘Swap It, Don’t Stop It’ assume everyone is motivated by weight loss and improved health. The challenge remains to achieve significant dietary changes at a population level to improve health, and it would appear there is benefit to integrating personalised goal setting for discretionary food and beverage consumption into broader lifestyle interventions.^(1)^ Given high consumption of discretionary foods across the population, such interventions are likely to be relevant and beneficial to many, not just those who are overweight. Interventions with tailored goal setting approaches may prove to be more effective,^(46)^ however further research is needed.

Nutrition interventions can be personalised in various ways, to account for demographic, motivation, dietary intake, barriers to change and genomics.^(47)^ However in practice, tailoring to complex factors such as genomics is uncommon and interventions are often limited to simple demographic and brief dietary intake data.^(48)^ Interestingly, findings from this study did not uncover any consistent results across personality characteristics, nor did the findings suggest that discretionary intake and BMI were related to goal setting helpfulness, but rather tailoring to gender, age and possibly education level may be a more effective approach. Personalised nutrition interventions are commonly delivered through digital platforms,^(47)^ as digital interventions provide a widely accessible and cost effective mode of delivery.^(49)^ The results from this study suggest that personalisation based on minimal data inputs, such as age, gender, and education level, may be enough to make goal setting more helpful for participants and possibly improve the success of interventions.

The current study has several strengths. Specifically, we used validated scales and questionnaires, including questions from the CSIRO Healthy Diet Score survey^(6)^ to assess dietary intake and the BFI-10 to assess personality traits. Longer formats of both tools exist, but were not used to minimise participant burden.^(32)^ Another strength was that this study was one of the first to explore individual preferences for types of goals and goal setting in relation to reducing discretionary food and beverage intake. Moreover, few studies have explored preferences for goal setting parameters in relation to demographic characteristics. Finally, the survey sample was large enough to include multiple potential predictor variables into regression analysis; however, no statistical power calculation was performed, nor were multiple comparisons controlled for and as such we encourage readers to exert caution when interpreting the findings. The cross-sectional study design is also a known limitation as causation cannot be implied.^(50)^ Another limitation is the generalisability of the sample. Recruitment of participants through a database of individuals who have previously been involved in CSIRO nutrition research increases the likelihood that this population was more interested in health than the broader Australian population. Further, certain groups of people, for instance, those who are illiterate or do not have access to technology, tend to be underrepresented in survey studies,^(51)^ and in our survey people with university education were overrepresented. A failure to meet the statistical assumptions led to the removal of participants who identified as non-binary, meaning that 3.5% of the Australian adult population was not represented in our sample.^(52)^ Finally, there are known biases associated with self-reported dietary intake and weight data; however, adjustment factors were applied to the dietary data to partially account for misreporting, which is usually in the direction of under-reporting for discretionary foods and beverages.^(26)^

## Conclusions

This exploratory, cross-sectional study demonstrated that age, gender, and education were associated with the goal types and goal setting parameters preferred by Australians in relation to eating less discretionary food and beverages. Participants with stronger habit strength preferred short-term, gradual, and specific goals, as well as those that were assigned and collaboratively set, indicating that additional support for these individuals when setting up a programme might be needed. These findings could help to inform the development of dietary interventions that use goal setting as a technique to reduce discretionary food and beverage intake, and to support individuals to set goals that align with their preferences, potentially leading to greater engagement and more effective interventions.

## Supporting information

Mauch et al. supplementary material Mauch et al. supplementary material

## Data Availability

Data is available from the corresponding author upon request.

## Supplementary material

The supplementary material for this article can be found at https://doi.org/10.1017/jns.2025.10022
