# Supplementary material for: Understanding Australian adults’ preferences for setting goals to reduce unhealthy food and beverage intake: a cross-sectional study
Source: J Nutr Sci. 2025 Jul 16;14:e50. doi: 10.1017/jns.2025.10022 (PMC12278176; doi:10.1017/jns.2025.10022)
Supplement: Mauch et al. supplementary material [file S2048679025100220sup001.docx]

**Supplementary material**

**Supplemental File 1:** Consumer Insights Survey; Understanding Australia’s ‘junk’ food and beverage choices

**Supplemental File 2:** Multinomial logistic regression exploring goal helpfulness (*not at all* vs. *slightly or somewhat* helpful) by demographics factors and personality traits

**Supplemental File 3:** Multinomial (*not at all* vs. *very or extremely* helpful) and ordinal logistic regression exploring goal helpfulness by demographics factors and personality traits

**Supplemental File 1:** Consumer Insights Survey; Understanding Australia’s ‘junk’ food and beverage choices

This survey is a part of a study exploring how food choices and behaviours can differ between Australian individuals. This survey will ask some questions about your intake of unhealthy foods and drinks, your reasons for eating them, and what might motivate you to eat less of them. This project is being supported by CSIRO and Flinders University.

**Section 1: Discretionary food and drink intake**

**These questions ask about how often you usually eat discretionary or unhealthy foods and drinks and when you do eat them, how much. There are no right or wrong answers so just respond as best as you can.**

Looking at the following list of foods, select one food category that you feel you would get most benefit from eating less of?

- Chocolate and confectionary
- Sugar sweetened beverages (includes, soft drinks, juices, ice teas etc)
- Cakes and biscuits
- Savoury snacks (e.g. pretzels, crisps)
- Muesli and snack bars
- Ice cream
- Fried potato products (e.g. hot chips)
- Processed meats (e.g. ham, salami, sausage, bacon)
- Takeaway foods (e.g. burgers, pizza, hot dogs, battered chicken).
- Savoury pastries (pies, pasties, sausage rolls)
- Alcohol

How often do you usually have meals or snacks from take away food stores?

* This includes places like McDonalds, Hungry Jacks, Pizza Hut, KFC, Red Rooster, Fish/Chicken Shop or local take away food places and foods such as burgers, pizza, hot dogs, battered chicken or fish and chips.

a. each day

b. each week

c. each month

d. never

In total, how many times do you usually have meals or snacks from these take away food stores in the timeframe selected above?

* This includes places like McDonalds, Hungry Jacks, Pizza Hut, KFC, Red Rooster, Fish/Chicken Shop or local take away food places and foods such as burgers, pizza, hot dogs, battered chicken or fish and chips.

0.5 times 14 times

How often do you usually eat oven baked potato gems/chips/hash browns, hot chips/French fries, wedges or fried potatoes?

a. each day

b. each week

c. each month

d. never

In total, how many serves of potato gems/chips/hash browns, hot chips/French fries, wedges or fried potatoes do you usually eat in the timeframe selected above?

* 1 serve = 12 fried hot chips 60g potato gems/hash browns, or wedges. Your answer can be in whole or half serves (e.g. 1, 1.5, 2, etc)

0.5 serves 14 serves

How often do you usually eat savoury snacks such as crisps, pretzels or plain/flavoured crackers?

a. each day

b. each week

c. each month

d. never

In total, how many serves of savoury snacks such as crisps, pretzels or plain/flavoured crackers do you usually eat in the timeframe selected above?

* 1 serve = ½ snack size packet of crisps 30g of salty crackers or pretzels

0.5 serves 14 serves

How often do you usually have sweet biscuits/cakes/buns/muffins/doughnuts?

* Include both home-made and bought.

a. each day

b. each week

c. each month

d. never

In total, how many serves of sweet biscuits/cakes/buns/muffins/doughnuts do you usually eat in the timeframe selected above?

* 1 serve = 2-3 (35g) sweet biscuits 1 doughnut 1 slice (40g) of plain cake or sweet bun 1 small muffin

0.5 serves 14 serves

How often do you usually eat savoury pastries?

* This includes pies, pasties, sausage rolls, Kransky Dogs and frankfurters wrapped in pastry.

a. each day

b. each week

c. each month

d. never

In total, how many serves of pies or savoury pastries do you usually eat in the timeframe selected above?

* 1 serve = 1/4 (60g) commercial meat pies or pastie 1 party size pie or sausage roll

0.5 serves 14 serves

How often do you usually eat snack type bars?

* This includes muesli bars, fruit bars and breakfast cereal bars.

a. each day

b. each week

c. each month

d. never

In total, how many snack types bars do you usually eat in the timeframe selected above?

* This includes muesli bars, fruit bars and breakfast cereal bars. Your answer can be in whole or half serves (e.g. 1, 1.5, 2, etc)

0.5 serves 14 serves

How often do you usually have chocolate or lollies?

* Include all types of chocolate and both hard and soft lollies.

a. each day

b. each week

c. each month

d. never

In total, how many serves of chocolate or lollies do you usually eat in the timeframe selected above?

* 1 serve = ½ chocolate bar 4 pieces of chocolate (25g) 5-6 (40g) lollies

0.5 serves 14 serves

How often do you usually have ice-cream or ice-blocks?

* This includes ice-blocks, ice-cream in a bowl or ice-creams on a stick.

a. each day

b. each week

c. each month

d. never

In total, how many serves of ice-cream or ice-blocks do you usually eat in the timeframe selected above?

* 1 serve = 2 scoops (60g) ice-cream 1 stick ice-cream or ice-block

0.5 serves 14 serves

How often do you usually drink alcohol?

* Include beer, wine, spirits and ciders

a. each day

b. each week

c. each month

d. never

In total, how many alcoholic drinks do you usually have in the timeframe selected above?

* 1 serve = 200ml of wine, a stubbie or can of beer (400ml) 60ml spirits

0.5 serves 14 serves

How often do you usually have soft drink, cordial or sports drinks?

* Include all drinks with added sugar such as soft drinks, cordials, fruit drinks, vitamin waters, energy and sports drinks.

a. each day

b. each week

c. each month

d. never

In total, how much soft drink, cordial or sports drinks do you usually drink in the timeframe selected above?

* 1 serve = 1 can (375ml) of soft drink 1 bottle of sports drink 375ml of cordial or fruit drink

0.5 serves 14 serves

How often do you usually eat meat products?

* Include sausages, frankfurters, devon, fritz, ham, salami, hot dogs, hamburgers and chicken nuggets.

a. each day

b. each week

c. each month

d. never

In total, how many serves of processed meat products do you usually eat in the timeframe selected above?

* 1 serve = 2 slices (55g) processed meat such as ham, salami, devon or fritz 2 thin or 1½ thick (60g) sausages, frankfurters or hot dogs 1 hamburger pattie 3 (60g) chicken nuggets

0.5 serves 14 serves

How often do you usually eat starchy vegetables?

* Starchy vegetables include potatoes, corn, sweet potato, taro, cassava and legumes (e.g. baked beans, chickpeas and lentils). DO NOT include hot chips.

a. each day

b. each week

c. each month

d. never

In total, how many serves of starchy vegetables (NOT including hot chips) do you usually eat in the timeframe selected above?

* 1 serve of starchy vegetables = ½ medium potato/sweet potato/cassava/taro ½ cup mashed potato (hot chips NOT included) ½ cup baked beans, cooked dried or canned beans, peas or lentils ½ cup or ½ cob of sweet corn

0.5 serves 14 serves

How often do you usually eat salad vegetables?

* Salad vegetables include lettuce, cucumber, tomato etc.

a. each day

b. each week

c. each month

d. never

In total, how many serves of salad vegetables do you usually eat in the timeframe selected above?

* 1 serve of salad vegetables = 1 cup green leafy or raw salad vegetables 1 medium tomato

0.5 serves 14 serves

How often do you usually eat cooked vegetables?

* Include baked, roasted, steamed, fried, grilled and boiled green or orange vegetables (e.g. broccoli, spinach, carrots, pumpkin). DO NOT include starchy vegetables.

a. each day

b. each week

c. each month

d. never

In total, how many serves of cooked vegetables do you usually eat in the timeframe selected above?

* 1 serve of cooked vegetables = ½ cup cooked fresh or frozen green or orange vegetables ½ cup tinned vegetables 1 cup vegetable soup (e.g. vegetable or pumpkin soup)

0.5 serves 14 serves

How often would your evening or main meal include three or more different vegetables? Include cooked, raw and salad vegetables.

a. Always (all the time)

b. Usually (two thirds of the time)

c. sometimes (half of the time)

d. never

e. I don’t eat vegetables with my main meal

**Section 2: Reasons, risks, intentions and confidence**

**In Australia, discretionary foods and drinks are those that are high in energy, saturated fat, added sugars, salt and alcohol. We will use the term ‘discretionary foods’ throughout this survey when referring to any or all of the following foods and drinks:**

- Chocolate and confectionary
- Sugar sweetened beverages (includes, soft drinks, juices, ice teas etc)
- Cakes and biscuits
- Savoury snacks (e.g. pretzels, crisps)
- Muesli and snack bars
- Ice cream
- Fried potato products (e.g. hot chips)
- Processed meats (e.g. ham, salami, sausage, bacon)
- Takeaway foods (e.g. burgers, pizza, hot dogs, battered chicken).
- Savoury pastries (pies, pasties, sausage rolls)
- Alcohol

These next questions help us understand your habits around eating discretionary food. Please rate your agreement with each of the items.

Response scale: 1 = strongly disagree to 5 = strongly agree

Eating discretionary food is something…

- 1. I do frequently.
  2. I do automatically.
  3. I do without having to consciously remember.
  4. that makes me feel weird if I do not do it.
  5. I do without thinking.
  6. that would require effort not to do it.
  7. That belongs to my (daily, weekly, monthly) routine.
  8. I start doing before I realise I’m doing it.
  9. I would find hard not to do.
  10. I have no need to think about doing.
  11. That’s typically “me”.
  12. I have been doing for a long time.

**Risk perception**

These questions are about how much of a problem you think your intake of discretionary foods pose to your health.

Indicate how much you agree with the following statement:

I eat too much discretionary food

1 = Strongly disagree

2 = Disagree

3 = Neither agree nor disagree

4 = Agree

5 = Strongly agree

Compared with an average person of your sex and age, do you think you eat:

1 = Much less discretionary food

2 = Slightly less discretionary food

3 = About the same amount of discretionary food

4 = Slightly more discretionary food

5 = Much more discretionary food

How serious a problem is your intake of discretionary food **to you**?

1 = Not serious at all (can be ignored)

2 = Somewhat serious

3 = Moderately serious

4 = Very serious

5 = Extremely serious (life-threatening)

In general, for the average person of your sex and age, how serious a problem is eating too much discretionary food?

1 = Not serious at all (can be ignored)

2 = Somewhat serious

3 = Moderately serious

4 = Very serious

5 = Extremely serious (life-threatening)

How would you rate your general health status?

1. Very good
2. Quite good
3. Neither good nor poor
4. Quite poor
5. Poor

**Intentions**

Next we’d like to know about your intentions ~~and plans~~, if any, to change your eating habits.

In the next month, do you intend to cut back on discretionary food?

1 = Don’t intend to at all

2 = Somewhat intend

3 = Moderately intend

4 = Strongly intend

**Confidence**

This question helps us to understand a little more about how you feel about your ability to change your eating habits.

*Action self-efficacy*

How confident are you that you could eat less discretionary food in the next month?

1 = Not at all confident

2 = Somewhat confident

3 = Moderately confident

4 = Extremely confident

**Section 3: Goal setting**

**These questions will help us gain insight into what you have tried before, and what might work in the future to help you to eat less discretionary foods. If you do not believe you need to eat less discretionary food and drinks, please skip this section.**

How many times have you tried to eat less discretionary food?

1. I haven’t ever tried
2. 1-2 times
3. 3-4 times
4. 5-6 times
5. 7 or more

When you have tried to eat less discretionary food did you use goal setting to help achieve this?

1. Never
2. Occasionally
3. Sometimes
4. Often
5. Always

How successful do you feel you were in achieving your goal of eating less discretionary food?

| Not very successful at all | Not successful | Somewhat successful | Successful | Very successful |
| --- | --- | --- | --- | --- |
| 1 | 2 | 3 | 4 | 5 |

Regardless if you have set a goal in the past or not, describe what a goal for eating less discretionary food might look like for you?

**_________________________________________________________________________**

*Please answer the next set of questions using the scale*

For the next set of questions, consider how you might make changes to your diet, and how helpful the following strategies would be to do so.

1. Setting short term goals. For example, eating less discretionary food for a week.

| Not at all helpful | Slightly helpful | Somewhat helpful | Very helpful | Extremely helpful |
| --- | --- | --- | --- | --- |
| 1 | 2 | 3 | 4 | 5 |

1. Setting long term goals. For example, eating less discretionary food for a month.

| Not at all helpful | Slightly helpful | Somewhat helpful | Very helpful | Extremely helpful |
| --- | --- | --- | --- | --- |
| 1 | 2 | 3 | 4 | 5 |

1. Eliminating discretionary foods all together. For example, eliminating all types of discretionary foods from my diet.

| Not at all helpful | Slightly helpful | Somewhat helpful | Very helpful | Extremely helpful |
| --- | --- | --- | --- | --- |
| 1 | 2 | 3 | 4 | 5 |

1. Gradually eating less discretionary food. For example, eating one less serve of discretionary food this week, then eating two less serves of discretionary food the following week.

| Not at all helpful | Slightly helpful | Somewhat helpful | Very helpful | Extremely helpful |
| --- | --- | --- | --- | --- |
| 1 | 2 | 3 | 4 | 5 |

1. Focusing on eating less of a particular type of discretionary food. For example, if your goal is to eat less discretionary foods, focusing on sugar sweetened beverages only.

| Not at all helpful | Slightly helpful | Somewhat helpful | Very helpful | Extremely helpful |
| --- | --- | --- | --- | --- |
| 1 | 2 | 3 | 4 | 5 |

1. Focusing on eating less of all discretionary foods, which includes all discretionary foods and drinks.

| Not at all helpful | Slightly helpful | Somewhat helpful | Very helpful | Extremely helpful |
| --- | --- | --- | --- | --- |
| 1 | 2 | 3 | 4 | 5 |

1. Setting goals for specific eating occasions. For example, if your goal is to eat less discretionary foods, eating less discretionary snacks at morning tea.

| Not at all helpful | Slightly helpful | Somewhat helpful | Very helpful | Extremely helpful |
| --- | --- | --- | --- | --- |
| 1 | 2 | 3 | 4 | 5 |

1. Having someone else (such as a health professional) set my goals for me to eat less discretionary foods.

| Not at all helpful | Slightly helpful | Somewhat helpful | Very helpful | Extremely helpful |
| --- | --- | --- | --- | --- |
| 1 | 2 | 3 | 4 | 5 |

1. Setting my own goals for eating less discretionary food.

| Not at all helpful | Slightly helpful | Somewhat helpful | Very helpful | Extremely helpful |
| --- | --- | --- | --- | --- |
| 1 | 2 | 3 | 4 | 5 |

1. Working with a health professional to set goals together, about eating less discretionary food.

| Not at all helpful | Slightly helpful | Somewhat helpful | Very helpful | Extremely helpful |
| --- | --- | --- | --- | --- |
| 1 | 2 | 3 | 4 | 5 |

1. Receiving regular feedback on my progress in reaching my goals for eating less discretionary food.

| Not at all helpful | Slightly helpful | Somewhat helpful | Very helpful | Extremely helpful |
| --- | --- | --- | --- | --- |
| 1 | 2 | 3 | 4 | 5 |

1. If you answered very helpful or extremely helpful to the question above, how often would you want to receive feedback?

More than once a day

Once a day

Once every few days

Once per week

Once every 2 weeks

Once a month

1. Tracking or recording my intake of discretionary foods. For example, recording my daily intake discretionary foods into a food diary.

| Not at all helpful | Slightly helpful | Somewhat helpful | Very helpful | Extremely helpful |
| --- | --- | --- | --- | --- |
| 1 | 2 | 3 | 4 | 5 |

1. Receiving reminders to track my intake of discretionary foods. For example, receiving reminder text messages.

| Not at all helpful | Slightly helpful | Somewhat helpful | Very helpful | Extremely helpful |
| --- | --- | --- | --- | --- |
| 1 | 2 | 3 | 4 | 5 |

1. Receiving additional feedback, when I am finding it difficult to reach my eat less discretionary food goal.

| Not at all helpful | Slightly helpful | Somewhat helpful | Very helpful | Extremely helpful |
| --- | --- | --- | --- | --- |
| 1 | 2 | 3 | 4 | 5 |

1. Receiving additional feedback, when I am going well to reach my eat less discretionary food goal.

| Not at all helpful | Slightly helpful | Somewhat helpful | Very helpful | Extremely helpful |
| --- | --- | --- | --- | --- |
| 1 | 2 | 3 | 4 | 5 |

**Section 4: Barriers and outcomes**

What do you think are the main barriers to you eating less discretionary food?

1. Family discouraging or unsupportive
2. Friends discouraging or unsupportive
3. People at work discouraging or unsupportive
4. Not knowing what changes to make
5. Not knowing how to cook more healthy foods
6. Lack of choice of healthy foods in canteens and restaurants
7. Lack of choice healthy foods in places where you do your main shop
8. Healthy foods are too expensive
9. Don’t like the taste/don’t enjoy healthy foods
10. Lack of willpower
11. Other (e.g. shift work and lack of time)
12. None of these – nothing preventing me from eating more healthy

**Outcome expectancies:**

What do you think will be the outcomes for yourself if you ate less discretionary food generally?

Indicate how much you agree with each statement, and how important each outcome is to you.

1 = Not at all true

2 = Somewhat true

3 = Mostly true

4 = Exactly true

1 = Not at all important

2 = Only slightly important

3 = Important

4 = Extremely important

**Positive outcomes:**

I would feel more attractive

How important is your appearance **to you**?

I would control my weight

How important is your weight **to you**?

The food I eat won’t taste as good

How important is the taste of food **to you**?

I would reduce my risk of some illnesses and diseases

How important is your risk of illness **to you**?

I would feel healthier

How important is how you feel in terms of health, **to you**?

I would have to make more effort to select and prepare food

How important is the effort you put into food **to you?**

I would have to spend more time preparing food

How important is the time you spend on preparing food **to you?**

My quality of life would be lower

How important is your quality of life **to you?**

I would feel hungrier

How important are your feelings of hunger and fullness **to you**?

I would be helping the environment

How important is the environment **to you**?

I would save money

How important is saving money **to you**?

My family would be pleased with the change

How important is what your family thinks **to you**?

It would harm my social life

How important is your social life **to you**?

I would have to change my habits

How important are your current habits **to you**?

**Section 5: About you**

*Please answer the next set of questions using the scale*

Personality has been shown to play a role in our behaviour around eating. These questions will help us explore your key personality traits.

How well do the following statements describe your personality?

I see myself a someone who…

1. … is reserved

| Strongly disagree | Disagree | Neutral | Agree | Strongly agree |
| --- | --- | --- | --- | --- |
| 1 | 2 | 3 | 4 | 5 |

1. …is generally trusting

| Strongly disagree | Disagree | Neutral | Agree | Strongly agree |
| --- | --- | --- | --- | --- |
| 1 | 2 | 3 | 4 | 5 |

1. …tends to be lazy

| Strongly disagree | Disagree | Neutral | Agree | Strongly agree |
| --- | --- | --- | --- | --- |
| 1 | 2 | 3 | 4 | 5 |

1. …is relaxed, handles stress well

| Strongly disagree | Disagree | Neutral | Agree | Strongly agree |
| --- | --- | --- | --- | --- |
| 1 | 2 | 3 | 4 | 5 |

1. …has few artistic interests

| Strongly disagree | Disagree | Neutral | Agree | Strongly agree |
| --- | --- | --- | --- | --- |
| 1 | 2 | 3 | 4 | 5 |

1. …is outgoing, sociable

| Strongly disagree | Disagree | Neutral | Agree | Strongly agree |
| --- | --- | --- | --- | --- |
| 1 | 2 | 3 | 4 | 5 |

1. …tends to find fault with others

| Strongly disagree | Disagree | Neutral | Agree | Strongly agree |
| --- | --- | --- | --- | --- |
| 1 | 2 | 3 | 4 | 5 |

1. …does a thorough job

| Strongly disagree | Disagree | Neutral | Agree | Strongly agree |
| --- | --- | --- | --- | --- |
| 1 | 2 | 3 | 4 | 5 |

1. …gets nervous easily

| Strongly disagree | Disagree | Neutral | Agree | Strongly agree |
| --- | --- | --- | --- | --- |
| 1 | 2 | 3 | 4 | 5 |

1. …has an active imagination

| Strongly disagree | Disagree | Neutral | Agree | Strongly agree |
| --- | --- | --- | --- | --- |
| 1 | 2 | 3 | 4 | 5 |

**Section 3: About You**

**This final section, is asking for some information about yourself.**

What is your gender identity?

1. Male
2. Female
3. Non-binary
4. Gender not listed

What is your age in years?

1. 18 – 19 years
2. 20 – 24 years
3. 25 - 29 years
4. 30 – 34 years
5. 35 – 39 years
6. 40 – 44 years
7. 45 – 49 years
8. 50 – 54 years
9. 55 – 59 years
10. 60 – 64 years
11. 65 – 69 years
12. 70 – 74 years
13. 75 – 79 years
14. 80 – 84 years
15. 85 – 89 years
16. 90 – 94 years
17. 95 – 99 years

What country were you born in?

1. Australia
2. Other: ________________________

What is your post code?

___________________

What is your weight in kg? e.g. 74kg

___________________

What is your height in cm? e.g. 164cm

________________

What is your highest level of education?

1. Less than high school
2. Finished high school (Year 12 or equivalent)
3. Certificate level (TAFE)
4. Diploma level
5. Bachelor’s degree
6. Postgraduate degree (honours, masters, doctorate, PhD).

**Thank you for taking the time to complete this survey. To go into the draw to win one of seven $50 Coles Group vouchers, please email the following address [study email address]**

**Supplemental File 2:** Multinomial logistic regression exploring goal helpfulness *(not at all* vs. *slightly or somewhat helpful*) by demographics factors and personality traits

**Supplemental File 2, Table 1: Multinomial logistic regression investigating predictors of short-term goal helpfulness**

|  | **Variables** | **B (SE)** | **OR** | **95% CI** | |
| --- | --- | --- | --- | --- | --- |
|  |  |  |  | **Lower** | **Upper** |
| Not at all vs. slightly or somewhat helpful | | | | | |
|  | Intercept | 5.38 (1.34) |  |  |  |
|  | Male (ref: female) | -0.20 (0.21) | 0.82 | 0.54 | 1.23 |
|  | Age 18-29 (ref:70+) | -0.03 (0.56) | 0.97 | 0.32 | 2.92 |
|  | Age 30-49 (ref:70+) | 0.15 (0.52) | 1.16 | 0.42 | 3.25 |
|  | Age 50-69 (ref:70+) | -0.16 (0.52) | 0.85 | 0.30 | 2.37 |
|  | No uni (ref: Uni) | 0.46 (0.22)* | 1.58 | 1.03 | 2.42 |
|  | BMI | -0.03 (0.01)* | 0.97 | 0.95 | 1.00 |
|  | Discretionary intake | -0.04 (0.02)* | 0.96 | 0.92 | 1.00 |
|  | Habit strength | 0.39 (0.14)** | 1.48 | 1.13 | 1.94 |
|  | Extraversion | -0.26 (0.07)*** | 0.77 | 0.67 | 0.89 |
|  | Agreeableness | 0.04 (0.06) | 1.04 | 0.92 | 1.18 |
|  | Conscientiousness | 0.02 (0.07) | 1.02 | 0.90 | 1.16 |
|  | Neuroticism | -0.03 (0.05) | 0.97 | 0.87 | 1.08 |
|  | Openness | -0.32 (0.07)*** | 0.73 | 0.63 | 0.84 |

*Abbreviations.* B, beta; SE, standard error; OR, odds ratio; CI, confidence interval; ref, reference; BMI, body mass index; * p<0.05; **p<0.01; ***p<0.001

**Supplemental File 2, Table 2: Multinomial logistic regression investigating predictors of elimination goal helpfulness**

|  | **Variables** | **B (SE)** | **OR** | **95% CI** | |
| --- | --- | --- | --- | --- | --- |
|  |  |  |  | **Lower** | **Upper** |
| Not at all vs. slightly or somewhat helpful | | | | | |
|  | Intercept | -0.04 (0.76) |  |  |  |
|  | Male (ref: female) | 0.57 (0.12)*** | 1.77 | 1.40 | 2.24 |
|  | Age 18-29 (ref:70+) | -0.23 (0.34) | 0.80 | 0.41 | 1.55 |
|  | Age 30-49 (ref:70+) | -0.28 (0.32) | 0.76 | 0.40 | 1.42 |
|  | Age 50-69 (ref:70+) | -0.20 (0.33) | 0.82 | 0.43 | 1.56 |
|  | No uni (ref: Uni) | -0.01 (0.12) | 0.99 | 0.79 | 1.25 |
|  | BMI | -0.00 (0.01) | 1.00 | 0.98 | 1.02 |
|  | Discretionary intake | -0.01 (0.02) | 0.99 | 0.96 | 1.02 |
|  | Habit strength | -0.02 (0.08) | 0.98 | 0.84 | 1.14 |
|  | Extraversion | -0.03 (0.04) | 0.97 | 0.90 | 1.05 |
|  | Agreeableness | 0.04 (0.04) | 1.04 | 0.97 | 1.12 |
|  | Conscientiousness | -0.02 (0.04) | 0.98 | 0.91 | 1.05 |
|  | Neuroticism | 0.01 (0.03) | 1.01 | 0.95 | 1.07 |
|  | Openness | -0.02 (0.04) | 0.98 | 0.91 | 1.06 |

*Abbreviations.* B, beta; SE, standard error; OR, odds ratio; CI, confidence interval; ref, reference; BMI, body mass index; * p<0.05; **p<0.01; ***p<0.001

**Supplemental File 2, Table 3: Multinomial logistic regression investigating predictors of food type goal (i.e. specific goal) helpfulness**

|  | **Variables** | **B (SE)** | **OR** | **95% CI** | |
| --- | --- | --- | --- | --- | --- |
|  |  |  |  | **Lower** | **Upper** |
| Not at all vs. slightly or somewhat helpful | | | | | |
|  | Intercept | 2.79 (1.31) |  |  |  |
|  | Male (ref: female) | 0.46 (0.22)* | 1.59 | 1.03 | 2.46 |
|  | Age 18-29 (ref:70+) | 0.78 (0.53) | 2.17 | 0.77 | 6.09 |
|  | Age 30-49 (ref:70+) | 0.64 (0.45) | 1.90 | 0.79 | 4.59 |
|  | Age 50-69 (ref:70+) | 0.15 (0.45) | 1.16 | 0.49 | 2.78 |
|  | No uni (ref: Uni) | -0.11 (0.20) | 0.89 | 0.61 | 1.32 |
|  | BMI | -0.00 (0.02) | 1.00 | 0.97 | 1.03 |
|  | Discretionary intake | -0.06 (0.02)** | 0.94 | 0.90 | 0.98 |
|  | Habit strength | 0.24 (0.13) | 1.27 | 0.98 | 1.65 |
|  | Extraversion | -0.09 (0.07) | 0.92 | 0.80 | 1.06 |
|  | Agreeableness | -0.03 (0.06) | 0.97 | 0.86 | 1.10 |
|  | Conscientiousness | -0.04 (0.07) | 0.96 | 0.84 | 1.09 |
|  | Neuroticism | 0.06 (0.05) | 1.06 | 0.96 | 1.18 |
|  | Openness | -0.18 (0.07)** | 0.84 | 0.73 | 0.96 |

*Abbreviations.* B, beta; SE, standard error; OR, odds ratio; CI, confidence interval; ref, reference; BMI, body mass index; * p<0.05; **p<0.01; ***p<0.001

**Supplemental File 2, Table 4: Multinomial logistic regression investigating predictors of eating occasion goal helpfulness**

|  | **Variables** | **B (SE)** | **OR** | **95% CI** | |
| --- | --- | --- | --- | --- | --- |
|  |  |  |  | **Lower** | **Upper** |
| Not at all vs. slightly or somewhat helpful | | | | | |
|  | Intercept | 3.68 (1.16) |  |  |  |
|  | Male (ref: female) | -0.19 (0.18) | 0.83 | 0.58 | 1.18 |
|  | Age 18-29 (ref:70+) | 1.12 (0.46)** | 3.07 | 1.26 | 7.51 |
|  | Age 30-49 (ref:70+) | 0.88 (0.40)** | 2.42 | 1.10 | 5.33 |
|  | Age 50-69 (ref:70+) | 0.49 (0.41) | 1.64 | 0.74 | 3.62 |
|  | No uni (ref: Uni) | -0.23 (0.17) | 0.80 | 0.57 | 1.12 |
|  | BMI | 0.01 (0.01) | 1.01 | 0.98 | 1.03 |
|  | Discretionary intake | -0.01 (0.02) | 0.99 | 0.95 | 1.03 |
|  | Habit strength | 0.12 (0.12) | 1.13 | 0.90 | 1.43 |
|  | Extraversion | -0.13 (0.06)* | 0.88 | 0.77 | 0.99 |
|  | Agreeableness | -0.04 (0.06) | 0.96 | 0.86 | 1.07 |
|  | Conscientiousness | -0.02 (0.06) | 0.98 | 0.88 | 1.09 |
|  | Neuroticism | -0.05 (0.05) | 0.96 | 0.87 | 1.05 |
|  | Openness | -0.24 (0.06)*** | 0.79 | 0.70 | 0.89 |

*Abbreviations.* B, beta; SE, standard error; OR, odds ratio; CI, confidence interval; ref, reference; BMI, body mass index; * p<0.05; **p<0.01; ***p<0.001

**Supplemental File 2, Table 5: Multinomial logistic regression investigating predictors of assigned goal helpfulness**

|  | **Variables** | **B (SE)** | **OR** | **95% CI** | |
| --- | --- | --- | --- | --- | --- |
|  |  |  |  | **Lower** | **Upper** |
| Not at all vs. slightly or somewhat helpful | | | | | |
|  | Intercept | -0.03 (0.83) |  |  |  |
|  | Male (ref: female) | 0.23 (0.13) | 1.26 | 0.97 | 1.64 |
|  | Age 18-29 (ref:70+) | 0.84 (0.35)* | 2.31 | 1.15 | 4.61 |
|  | Age 30-49 (ref:70+) | 0.42 (0.32) | 1.53 | 0.81 | 2.86 |
|  | Age 50-69 (ref:70+) | 0.06 (0.32) | 1.06 | 0.56 | 2.00 |
|  | No uni (ref: Uni) | -0.24 (0.13) | 0.79 | 0.61 | 1.01 |
|  | BMI | 0.01 (0.01) | 1.01 | 0.99 | 1.03 |
|  | Discretionary intake | -0.01 (0.02) | 0.99 | 0.96 | 1.02 |
|  | Habit strength | 0.19 (0.09)* | 1.20 | 1.02 | 1.42 |
|  | Extraversion | -0.08 (0.05) | 0.92 | 0.85 | 1.01 |
|  | Agreeableness | 0.07 (0.04) | 1.07 | 0.99 | 1.16 |
|  | Conscientiousness | -0.11 (0.04)** | 0.90 | 0.83 | 0.97 |
|  | Neuroticism | 0.05 (0.03) | 1.05 | 0.99 | 1.12 |
|  | Openness | -0.02 (0.04) | 0.98 | 0.90 | 1.07 |

*Abbreviations.* B, beta; SE, standard error; OR, odds ratio; CI, confidence interval; ref, reference; BMI, body mass index; * p<0.05; **p<0.01; ***p<0.001

**Supplemental File 2, Table 6: Multinomial logistic regression investigating predictors of collaborative goal helpfulness**

|  | **Variables** | **B (SE)** | **OR** | **95% CI** | |  |
| --- | --- | --- | --- | --- | --- | --- |
|  |  |  |  | **Lower** | **Upper** |  |
| Not at all vs. slightly or somewhat helpful | | | | | | |
|  | Intercept | 0.52 (0.99) |  |  |  |  |
|  | Male (ref: female) | 0.08 (0.16) | 1.09 | 0.80 | 1.48 |  |
|  | Age 18-29 (ref:70+) | 1.46 (0.39)*** | 4.33 | 2.00 | 9.35 |  |
|  | Age 30-49 (ref:70+) | 1.03 (0.33)** | 2.80 | 1.46 | 5.36 |  |
|  | Age 50-69 (ref:70+) | 0.40 (0.33) | 1.49 | 0.78 | 2.86 |  |
|  | No uni (ref: Uni) | -0.06 (0.15) | 0.95 | 0.70 | 1.28 |  |
|  | BMI | 0.01 (0.01) | 1.01 | 0.99 | 1.04 |  |
|  | Discretionary intake | -0.01 (0.02) | 0.99 | 0.95 | 1.03 |  |
|  | Habit strength | 0.06 (0.10) | 1.06 | 0.87 | 1.29 |  |
|  | Extraversion | -0.08 (0.05) | 0.92 | 0.83 | 1.03 |  |
|  | Agreeableness | 0.03 (0.05) | 1.03 | 0.94 | 1.13 |  |
|  | Conscientiousness | -0.07 (0.05) | 0.93 | 0.85 | 1.03 |  |
|  | Neuroticism | 0.03 (0.04) | 1.03 | 0.96 | 1.12 |  |
|  | Openness | -0.03 (0.05) | 0.97 | 0.88 | 1.07 |  |

*Abbreviations.* B, beta; SE, standard error; OR, odds ratio; CI, confidence interval; ref, reference; BMI, body mass index; * p<0.05; **p<0.01; ***p<0.001

**Supplemental File 3:** Multinomial (*not at all* vs. *very or extremely* helpful) and ordinal logistic regression exploring goal helpfulness by demographics factors and personality traits

**Supplemental File 3, Table 1: Logistic regression investigating predictors of short-term and long-term goal helpfulness.**

|  | **Short-term goals^a^ (Not at all vs. very or extremely helpful)** | | | |  | **Long-term goals^b^** | | | | |  |
| --- | --- | --- | --- | --- | --- | --- | --- | --- | --- | --- | --- |
| **Variables** | **B (SE)** | **OR** | **95% CI** | |  | **B (SE)** | **OR** | **95% CI** | | |  |
|  |  |  | **Lower** | **Upper** | **p-value** |  |  | **Lower** | **Upper** | | **p-value** |
| Intercept | 2.82 (1.40) |  |  |  | .044 |  |  |  | |  |  |
| Male (ref: female) | -0.34 (0.22) | 0.71 | 0.47 | 1.09 | .119 | 0.34 (0.10)*** | 1.41 | 1.17 | | 1.70 | .000 |
| Age 18-29 (ref:70+) | 0.72 (0.62) | 2.06 | 0.61 | 6.91 | .245 | 0.48 (0.28) | 1.62 | 0.93 | | 2.81 | .088 |
| Age 30-49 (ref:70+) | 0.89 (0.58) | 2.43 | 0.78 | 7.63 | .127 | 0.60 (0.27)* | 1.81 | 1.08 | | 3.05 | .025 |
| Age 50-69 (ref:70+) | 0.61 (0.58) | 1.83 | 0.59 | 5.75 | .298 | 0.85 (0.27)** | 2.34 | 1.38 | | 3.96 | .002 |
| No uni (ref: Uni) | 0.65 (0.22)** | 1.92 | 1.23 | 2.97 | .004 | 0.16 (0.09) | 1.18 | 0.98 | | 1.41 | .080 |
| BMI | -0.03 (0.01) | 0.98 | 0.95 | 1.00 | .074 | -0.01 (0.01) | 0.99 | 0.98 | | 1.01 | .289 |
| Discretionary intake | -0.04 (0.02) | 0.97 | 0.92 | 1.01 | .130 | -0.02 (0.01) | 0.98 | 0.96 | | 1.01 | .140 |
| Habit strength | 0.34 (0.14)* | 1.40 | 1.06 | 1.86 | .018 | -0.18 (0.06)** | 0.84 | 0.74 | | 0.95 | .005 |
| Extraversion | -0.22 (0.08)** | 0.80 | 0.69 | 0.93 | .003 | -0.05 (0.03) | 0.95 | 0.89 | | 1.02 | .150 |
| Agreeableness | 0.17 (0.07)* | 1.18 | 1.04 | 1.34 | .013 | 0.05 (0.03) | 1.05 | 1.00 | | 1.12 | .072 |
| Conscientiousness | 0.07 (0.07) | 1.07 | 0.94 | 1.23 | .288 | 0.02 (0.03) | 1.02 | 0.96 | | 1.08 | .575 |
| Neuroticism | -0.06 (0.06) | 0.94 | 0.85 | 1.05 | .291 | -0.06 (0.02)* | 0.94 | 0.90 | | 0.99 | .012 |
| Openness | -0.31 (0.08)*** | 0.73 | 0.63 | 0.85 | .000 | -0.04 (0.03) | 0.96 | 0.90 | | 1.02 | .157 |

*Abbreviations.* B, beta; SE, standard error; OR, odds ratio; CI, confidence interval; ref, reference; BMI, body mass index; ^a^, Multinomial logistic regression; ^b^, Ordinal logistic regression; * p<0.05; **p<0.01; ***p<0.001

**Supplemental File 3, Table 2: Logistic regression investigating predictors of elimination and gradual goal helpfulness.**

|  | **Elimination goals ^a^  (Not at all vs. very or extremely helpful)** | | | |  | **Gradual goals ^b^** | | | |  |
| --- | --- | --- | --- | --- | --- | --- | --- | --- | --- | --- |
| **Variables** | **B (SE)** | **OR** | **95% CI** | |  | **B (SE)** | **OR** | **95% CI** | |  |
|  |  |  | **Lower** | **Upper** | **p-value** |  |  | **Lower** | **Upper** | **p-value** |
| Intercept | -2.00 (1.00) |  |  |  | .046 |  |  |  |  |  |
| Male (ref: female) | 0.82 (0.15)*** | 2.26 | 1.70 | 3.02 | .000 | 0.00 (0.09) | 1.00 | 0.83 | 1.20 | .997 |
| Age 18-29 (ref:70+) | -0.04 (0.49) | 0.96 | 0.37 | 2.50 | .940 | 0.54 (0.28) | 1.72 | 0.99 | 2.96 | .053 |
| Age 30-49 (ref:70+) | 0.29 (0.46) | 1.34 | 0.55 | 3.28 | .526 | 0.14 (0.26) | 1.16 | 0.69 | 1.93 | .583 |
| Age 50-69 (ref:70+) | 0.58 (0.46) | 1.78 | 0.72 | 4.39 | .209 | 0.18 (0.27) | 1.20 | 0.71 | 2.02 | .493 |
| No uni (ref: Uni) | 0.38 (0.14)** | 1.47 | 1.11 | 1.94 | .007 | 0.27 (0.09)** | 1.30 | 1.09 | 1.56 | .004 |
| BMI | -0.00 (0.01) | 1.00 | 0.98 | 1.02 | .872 | 0.00 (0.01) | 1.00 | 0.99 | 1.02 | .769 |
| Discretionary intake | 0.02 (0.02) | 1.02 | 0.99 | 1.05 | .269 | 0.00 (0.01) | 1.00 | 0.98 | 1.02 | .966 |
| Habit strength | -0.06 (0.10) | 0.94 | 0.77 | 1.14 | .519 | 0.13 (0.06)* | 1.14 | 1.01 | 1.29 | .033 |
| Extraversion | 0.02 (0.05) | 1.02 | 0.92 | 1.13 | .660 | 0.00 (0.03) | 1.00 | 0.94 | 1.07 | .904 |
| Agreeableness | 0.02 (0.05) | 1.02 | 0.93 | 1.11 | .736 | 0.04 (0.03) | 1.05 | 0.99 | 1.11 | .131 |
| Conscientiousness | 0.02 (0.05) | 1.02 | 0.93 | 1.12 | .720 | 0.07 (0.03)* | 1.07 | 1.01 | 1.14 | .014 |
| Neuroticism | -0.04 (0.04) | 0.96 | 0.89 | 1.04 | .303 | 0.06 (0.02)* | 1.06 | 1.02 | 1.11 | .010 |
| Openness | 0.03 (0.05) | 1.03 | 0.94 | 1.13 | .552 | -0.02 (0.03) | 0.98 | 0.92 | 1.04 | .518 |

*Abbreviations.* B, beta; SE, standard error; OR, odds ratio; CI, confidence interval; ref, reference; BMI, body mass index; ^a^, Multinomial logistic regression; ^b^, Ordinal logistic regression; * p<0.05; **p<0.01; ***p<0.001

**Supplemental File 3, Table 3: Logistic regression investigating predictors of food type goal (i.e., specific goal), all food goals (i.e. broad goals), and eating occasion goal helpfulness.**

|  | **Food type goals i.e., specific goals (Not at all vs. very or extremely helpful)^a^** | | | | **All food goals i.e., broad goals ^b^** | | | | **Eating occasion goals (Not at all vs. very or extremely helpful) ^a^** | | | |
| --- | --- | --- | --- | --- | --- | --- | --- | --- | --- | --- | --- | --- |
| **Variables** | **OR** | **95% CI** | | **p-value** | **OR** | **95% CI** | | **p-value** | **OR** | **95% CI** | | **p-value** |
|  |  | **Lower** | **Upper** |  |  | **Lower** | **Upper** |  |  | **Lower** | **Upper** |  |
| Intercept |  |  |  | .488 |  |  |  |  |  |  |  | .062 |
| Male (ref: female) | 1.66 | 1.07 | 2.58 | .024* | 1.47 | 1.22 | 1.77 | .000*** | 0.70 | 0.49 | 1.01 | .057 |
| Age 18-29 (ref:70+) | 4.04 | 1.39 | 11.73 | .010* | 1.60 | 0.93 | 2.78 | .092 | 3.79 | 1.49 | 9.60 | .005** |
| Age 30-49 (ref:70+) | 2.52 | 1.00 | 6.35 | .050 | 1.85 | 1.10 | 3.12 | .020* | 2.48 | 1.08 | 5.69 | .032* |
| Age 50-69 (ref:70+) | 1.43 | 0.57 | 3.59 | .444 | 2.22 | 1.31 | 3.75 | .003** | 1.94 | 0.85 | 4.45 | .118 |
| No uni (ref: Uni) | 0.72 | 0.48 | 1.06 | .097 | 1.31 | 1.09 | 1.57 | .004** | 0.88 | 0.62 | 1.25 | .481 |
| BMI | 1.00 | 0.97 | 1.03 | .990 | 1.00 | 0.99 | 1.02 | .455 | 1.00 | 0.98 | 1.03 | .929 |
| Discretionary intake | 0.96 | 0.92 | 1.00 | .065 | 0.98 | 0.96 | 1.00 | .117 | 0.99 | 0.95 | 1.03 | .677 |
| Habit strength | 1.35 | 1.04 | 1.76 | .023* | 0.89 | 0.79 | 1.01 | .063 | 1.04 | 0.82 | 1.32 | .761 |
| Extraversion | 0.97 | 0.84 | 1.11 | .629 | 0.97 | 0.91 | 1.03 | .296 | 0.88 | 0.78 | 1.00 | .056 |
| Agreeableness | 1.00 | 0.88 | 1.14 | .987 | 1.00 | 0.95 | 1.06 | .975 | 1.10 | 0.98 | 1.23 | .114 |
| Conscientiousness | 0.99 | 0.87 | 1.13 | .887 | 0.99 | 0.93 | 1.04 | .601 | 1.02 | 0.91 | 1.14 | .790 |
| Neuroticism | 1.08 | 0.97 | 1.19 | .168 | 1.00 | 0.96 | 1.05 | .869 | 0.98 | 0.89 | 1.07 | .599 |
| Openness | 0.89 | 0.77 | 1.01 | .077 | 0.99 | 0.93 | 1.05 | .684 | 0.80 | 0.71 | 0.91 | .001** |

*Abbreviations.* B, beta; SE, standard error; OR, odds ratio; CI, confidence interval; ref, reference; BMI, body mass index; ^a^, Multinomial logistic regression; ^b^, Ordinal logistic regression; * p<0.05; **p<0.01; ***p<0.001

**Supplemental File 3, Table 4: Logistic regression investigating predictors of assigned goal, self-set goal, and collaborative goal helpfulness.**

| **Assigned goals (Not at all vs. very or extremely helpful)** **^a^** | | | |  | **Self-set goals ^b^** | | |  | **Collaborative goals ^a^** | | |  |
| --- | --- | --- | --- | --- | --- | --- | --- | --- | --- | --- | --- | --- |
| **Variables** | **OR** | **95% CI** | | **p-value** | **OR** | **95% CI** | | **p-value** | **OR** | **95% CI** | | **p-value** |
|  |  | **Lower** | **Upper** |  |  | **Lower** | **Upper** |  |  | **Lower** | **Upper** |  |
| Intercept |  |  |  | .003 |  |  |  |  |  |  |  | .020 |
| Male (ref: female) | 1.22 | 0.92 | 1.63 | .170 | 1.23 | 1.02 | 1.49 | .031* | 0.98 | 0.71 | 1.35 | .894 |
| Age 18-29 (ref:70+) | 5.41 | 2.01 | 14.52 | .001** | 1.89 | 1.08 | 3.29 | .025* | 15.25 | 5.66 | 41.08 | .000*** |
| Age 30-49 (ref:70+) | 3.51 | 1.37 | 8.96 | .009** | 1.83 | 1.09 | 3.10 | .023* | 7.48 | 3.03 | 18.46 | .000*** |
| Age 50-69 (ref:70+) | 2.66 | 1.03 | 6.83 | .042* | 2.04 | 1.20 | 3.47 | .009** | 4.00 | 1.62 | 9.88 | .003** |
| No uni (ref: Uni) | 1.20 | 0.91 | 1.57 | .197 | 0.90 | 0.75 | 1.09 | .282 | 1.09 | 0.80 | 1.48 | .572 |
| BMI | 0.99 | 0.97 | 1.01 | .481 | 0.98 | 0.97 | 0.99 | .001** | 1.02 | 1.00 | 1.04 | .091 |
| Discretionary intake | 1.01 | 0.98 | 1.05 | .409 | 0.99 | 0.97 | 1.01 | .396 | 0.98 | 0.95 | 1.02 | .392 |
| Habit strength | 1.38 | 1.14 | 1.66 | .001** | 0.73 | 0.64 | 0.82 | .000*** | 1.24 | 1.01 | 1.53 | .037* |
| Extraversion | 1.01 | 0.91 | 1.11 | .916 | 0.98 | 0.92 | 1.05 | .584 | 0.97 | 0.87 | 1.08 | .570 |
| Agreeableness | 1.13 | 1.04 | 1.23 | .006** | 1.06 | 1.00 | 1.12 | .070 | 1.09 | 0.99 | 1.20 | .074 |
| Conscientiousness | 0.92 | 0.85 | 1.01 | .072 | 1.08 | 1.02 | 1.14 | .013* | 0.96 | 0.87 | 1.06 | .423 |
| Neuroticism | 1.05 | 0.98 | 1.13 | .149 | 0.94 | 0.89 | 0.98 | .006** | 1.06 | 0.98 | 1.15 | .145 |
| Openness | 1.01 | 0.92 | 1.11 | .825 | 1.05 | 0.99 | 1.12 | .094 | 0.98 | 0.88 | 1.08 | .658 |

*Abbreviations.* B, beta; SE, standard error; OR, odds ratio; CI, confidence interval; ref, reference; BMI, body mass index; ^a^, Multinomial logistic regression; ^b^, Ordinal logistic regression; * p<0.05; **p<0.01; ***p<0.001
